# Circ_0000020 elevates the expression of PIK3CA and facilitates the malignant phenotypes of glioma cells via targeting miR-142-5p

**DOI:** 10.1186/s12935-021-01767-5

**Published:** 2021-01-28

**Authors:** Xu Wang, Yaozu Zhu

**Affiliations:** Department of Neurosurgery, Xiangyang Central Hospital, Affiliated Hospital of Hubei University of Arts and Science, Jingzhou Street No. 136, Xiangyang, 441021 Hubei China

**Keywords:** Glioma, circ_0000020, miR-142-5p, PIK3CA

## Abstract

**Background:**

Multiple circular RNAs (circRNAs) have been recently described as crucial oncogenic factors or tumor suppressors. This study aimed to investigate the role of circ_0000020 in glioma progression.

**Methods:**

Circ_0000020 and miR-142-5p expressions in glioma samples were assessed through qRT-PCR, and then the association between pathological indexes and circ_0000020 expressions was analyzed. Functional experiment was performed with human glioma cell lines U251 and U87. Gain-of-function and loss-of-function models were established. CCK-8 assay was used to detect glioma cell proliferation. Transwell assay was used to examine glioma cell migration and invasion. The regulatory relationships among circ_0000020, miR-142-5p and phosphatidylinositol 3-kinase C (PIK3CA) were investigated by bioinformatics analysis, luciferase reporter assay, qRT-PCR and Western blot. In vivo tumorigenesis assay was performed with nude mice to further validate the demonstrations of in vitro experiments.

**Results:**

Circ_0000020 expression in glioma samples was remarkably increased compared with that in normal brain tissues and its high expression was associated with unfavorable pathological indexes. Circ_0000020 overexpression remarkably accelerated proliferation, migration and invasion of glioma cells. Accordingly, circ_0000020 knockdown suppressed the malignant phenotypes of glioma cells. Circ_0000020 overexpression significantly reduced miR-142-5p expression by sponging it, and circ_0000020 could enhance the expression of PIK3CA, which was a target gene of miR-142-5p.

**Conclusions:**

Circ_0000020 promotes glioma progression via miR-142-5p/PIK3CA axis.

## Background

Glioma accounts for 40–60% in all intracranial tumors [1]. Despite the continuous improvement of treatment strategies such as surgery, radiotherapy, and chemotherapy, the median survival time of patients is far from satisfactory [2, 3]. Clarifying the mechanism of glioma progression may provide clues for the diagnosis and treatment of this deadly disease.

Circular RNA (circRNA) is a type of RNA that has a closed-loop structure formed by reverse splicing and covalent bonding [4]. CircRNA has complex biological functions [5]. Accumulating studies authenticate that abnormal expression of circRNA is related to the occurrence and progression of human malignancies, gliomas included [6–8]. For example, circTTBK2 expression is increased in glioma tissues and cell lines, and its overexpression accelerates cancer cell proliferation, migration, and invasion [6]; circ_0074362 expression is dramatically up-regulated in glioma tissues, and its high expression is markedly related to the unfavorable pathological characteristics and poor prognosis of patients [7].

MicroRNAs (miRNAs) are an endogenous small non-coding RNA (ncRNAs) containing 18-25 nucleotides [9]. MiRNAs can completely or incompletely pair with the 3′-UTR of target mRNA, therefore leading to mRNA degradation or post-transcriptional translational inhibition [10]. MiRNAs are important players in cancer biology [11, 12]. MiR-142-5p expression is increased in colorectal cancer, and miR-142-5p enhances the cancer progression via targeting SDHB [13]. Besides, miR-142-5p blocks the progression of pancreatic cancer by targeting RAP1A [14]. Moreover, reportedly, miR-142-3p expression is decreased in gliomas, and miR-142-3p overexpression can remarkably repress glioma cell migration and invasion [15]. Instead, the role of miR-142-5p in glioma still await more investigations. Interestingly, in our preliminary work, bioinformatics analysis suggests that circ_0000020 can probably repress miR-142-5p, suggesting circ_0000020 may exert oncogenic functions in glioma via inhibiting miR-142-5p.

Phosphatidyl inositol kinase 3 catalytic subunit alpha (PIK3CA) gene is located on chromosome 3q26.3 and encodes a protein containing 1068 amino acid residues. PIK3CA encodes the p110 catalytic subunit of class I phosphatidylino-sitol 3-kinases (PI3Ks), namely PI3Kp110a [16]. PIK3CA mutations can not only strengthen the catalytic activity of PI3Ks, but also contribute to tumorigenesis [17, 18]. Importantly, previous studies report that PIK3CA expression is elevated in gliomas and it promotes glioma progression [19, 20].

In this work, we confirmed that circ_0000020 expression was elevated in glioma tissues and cell lines. The high expression of circ_0000020 was markedly associated with unfavorable clinicopathological indicators. Additionally, we demonstrated that circ_0000020 promoted the proliferation, migration and invasion of glioma cells via modulating miR-142-5p/PI3KCA axis. These results implied that circ_0000020 might be a biomarker and therapeutic target for gliomas.

## Materials and methods

### Tissue samples

Our study was ratified by the Research Ethics Committee of Xiangyang Central Hospital and obtained consent from all of the subjects involved. Tissue specimens (paired cancerous tissues and adjacent tissues) were from 50 randomly selected glioma patients, 29 males and 21 females included, who had undergone tumor resection in Xiangyang Central Hospital from 2015 to 2017. Adjacent tissues were the non-cancerous tissues of the same patient (about 3 cm away from the surgical margin). Before this study, the tissues were diagnosed and re-evaluated according to the World Health Organization (WHO) criteria by two experienced pathologists. All samples were stored in liquid nitrogen at − 196 °C until RNA extraction.

### Cell culture

Cell Center of Chinese Academy of Sciences (Shanghai, China) was the provider of normal human astrocyte (NHA) and glioma cell line (U87, SW1008, T98G, U251 and U138). Cells were cultivated in Dulbecco’s Modified Eagle’s Medium (DMEM, Gibco, Carlsbad, CA, USA) with 10% fetal bovine serum (FBS) (Thermo Fisher Scientific, MA, USA) and 100 U/mL penicillin, 100 μg/mL streptomycin (Hyclone, Logan, UT, USA) at 37 °C in 5% CO_2_.

### Cell transfection

pcDNA empty vector (Control), pcDNA-circ_0000020 (circ_0000020), siRNA normal control (si-NC), siRNA against circ_0000020 (si-circ_0000020), siRNA against PIK3CA (si-PIK3CA), miRNA control (miR NC, sense 5′-UUCUCCGAACGUGUCACGUTT-3′ and antisense 5′-ACGUGACACGUUCGGAGAATT-3′), inhibitor control (Inhibitor NC) (5′-CAGUACUUUUGUGUAGUACAA-3′), miR-142-5p inhibitors (5′-AGUAGUGCUUUCUACUUUAUG-3′) and miR-142-5p mimics (sense 5′-CAUAAAGUAGAAAGCACUACU-3′ and antisense 5′-UAGUGCUUUCUACUUUAUGUU-3′) were available from GenePharma (Shanghai, China). For about 5 × 10^5^ cells, 500 ng of plasmids, siRNAs or miRNAs were transfected employing 1.5 μL of Lipofectamine^®^ 3000 (Invitrogen, Carlsbad, CA, USA). 48 h after the transfection, the transfection efficiency was determined by quantitative real-time polymerase chain reaction (qRT-PCR), and then the cells were harvested for other experiments.

### qRT-PCR

Total RNA was obtained from glioma tissue and cells with RNAiso Plus reagent (Takara, Dalian, China) according to the manufacture’s instruction. The complementary DNA (cDNA) was synthesized with 1 μg of RNA as template using PrimeScript 1st Stand cDNA Synthesis Kit (Takara, Dalian, China). The total volume of PCR system was 30 μL, and each sample contained 300 ng cDNA. qRT-PCR was performed using SYBR Green PCR Master Mix (Applied Biosystems, Shanghai, China). The relative expressions of circ_0000020, PI3KCA and miR-142-5p were calculated through 2^−∆∆Ct^ method, with glyceraldehyde-3-phosphate dehydrogenase (GAPDH; for circ_0000020 and PI3KCA mRNA) or U6 (for miR-142-5p) as internal reference. The primer sequence information was listed in Table 1.

**Table 1:** qRT-PCR primer sequences

Name	Primer sequences
circ_0000020	Forward: 5′-GAGAGGATGTACGGCCAGAG-3′
	Reverse: 5′-AAACTTTCCGGAGCCTCTTC-3′
miR-142-5p	Forward: 5′-GGATCATAAAGTAGAAAA-3′
	Reverse: 5′-CAGTGTGTCGTGGAGT-3′
PIK3CA	Forward: 5′-CCACGACCATCATCAGGTGAA-3′
	Reverse: 5′-CCTCACGGAGATTCTAAAGT-3′
DDI2	Forward: 5′-CTCCGAGGTGACCTTTTCCC-3′
	Reverse: 5′-CTGTGAGAGGTCTTTCCGCA-3′
GAPDH	Forward: 5′-GAAGGTGAAGGTCGGAGTC-3′
	Reverse: 5′-GAAGATGGTGATGGGATTTC-3′
U6	Forward: 5′-CTCGCTTCGGCAGCACA-3′
	Reverse: 5′-AACGCTTCACGAATTTGCGT-3′

### Cell counting kit-8 (CCK-8) assay

Glioma cells were inoculated into the 96-well plate (2 × 10^3^ cells in each well). 6 wells were set for each group. After 12 h of culture, 10 μL CCK-8 reagent (Beyotime, Shanghai, China) was loaded into each well. A blank well only contained the medium and CCK-8 reagent. The culture was continued in the incubator for 4 h, and then the absorbance of the cells at 450 nm was recorded by a microplate reader. With this method, the absorbance of U87 and U251 cells was measured at 12, 24, 48, 72 and 96 h to plot the proliferation curve.

### Cell migration and invasion assay

Transwell assay was performed with Transwell chambers (Millipore, Billerica, USA). In migration assay, glioma cells were collected after the transfection, centrifuged at 100*g* for 3 min, and then resuspended in serum-free DMEM to a density of 1 × 10^5^ cells/mL. 200 μL of the cell suspension and 700 μL of complete medium with 10% FBS were dripped into the upper compartment and the lower compartment of each Transwell chamber (8 μm pore size, Corning, NY, USA), respectively. Then the cells were incubated in at 37 °C in 5% CO_2_ for 24 h. Then the cells on the upper surface of the membrane were gently wiped off with cotton swabs, and then the membrane was immersed in crystal violet solution, and stained for 30 min. After that, the cells were observed and photographed with a microscope (Olympus, Tokyo, Japan). For each chamber, 5 visual fields were selected randomly, and the cells were counted, and then the average was taken. In invasion assay, Matrigel (Clontech, Madison, WI, USA) was diluted, and used to cover the membrane. Other experimental steps were the same as in the migration experiment.

### Luciferase reporter assay

The sequence of circ_0000020 or PIK3CA 3′-UTR containing the predicted wild-type (WT) or mutant (Mut) miR-142-5p binding sites was inserted into the luciferase reporter vector pmirGLO (Promega, Madison, WI, USA) to generate the reporter vectors, namely WT-pmirGLO-circ_0000020, Mut-pmirGLO-circ_0000020, WT-pmirGLO-PIK3CA 3′ UTR, Mut1-pmirGLO-PIK3CA 3′ UTR, Mut2-pmirGLO-PIK3CA 3′ UTR, Mut3-pmirGLO-PIK3CA 3′ UTR, and Mut1&2&3-pmirGLO-PIK3CA 3′ UTR. U87 and U251 cells (4.5 × 10^4^ cells/mL) were inoculated in 48-well plates and cultured to 70% confluence. Then the reporter vectors were co-transfected with miR-142-5p mimics or miR NC into the glioma cells, respectively, with Lipofectamine^®^3000 (Invitrogen, Carlsbad, CA, USA). 48 h after the transfection, luciferase activity was detected using dual-luciferase reporter assay system (Promega, Madison, WI, USA) according to the manufacture’s instruction. Firefly luciferase activity was normalized to renilla luciferase activity.

### Western blot

After the cells were washed, RIPA lysis buffer (Beyotime, Shanghai, China) was used to extract the total protein. Then the total protein was separated by SDS-PAGE, followed by being electrically transferred to PVDF membrane (Millipore, Bedford, MA, USA). The PVDF membranes were blocked with 5% skim milk at room temperature for 1 h and washed with TBST 3 times, each time for 10 min. Then the membranes were incubated overnight with the primary antibodies Anti-PIK3CA (ab40776, Abcam, 1:1000) and anti-GAPDH (ab8245, Abcam, 1:1000) at 4 °C. After that, TBST was adopted to rinse the membrane 3 times, each time for 15 min, and then the membrane and the secondary antibody (Beyotime, Shanghai, China, 1:2000) were incubated at room temperature for 2 h before the membrane was rinsed with TBST three times again. After that, enhanced chemiluminescence kit (Millipore, Bedford, MA, USA) was utilized to develop the protein bands.

### In vivo tumorigenesis assay

4-week old female BALB/c athymic nude mice were used for animal experiments. All animal experiments were endorsed by the Animal Experimental Committee of Xiangyang Central Hospital. U87 cells transfected with si-NC or si-circ_0000020 were harvested, and resuspended in 200 μL of phosphate-buffered saline. Then the cells were subcutaneously injected into the left flank of the nude mice (n = 5 in each group, 5 × 10^6^ cells per mice). Tumor growth was examined every 5 d. 30 d later, the mice were euthanized and the tumors were resected and weighted. Lung metastasis model was established by injecting U87 cells into the caudal vein of the mice (n = 5 in each group, 5 × 10^6^ cells per mice). 14 d later, the mice were euthanized, and the lungs were harvested, and hematoxylin and eosin (H&E) staining was performed.

### Statistical analysis

All experiments were performed in triplicate (or more) and repeated for at least three times. The data were processed using SPSS 17.0 statistical software (SPSS Inc., Chicago, IL, USA). The results were expressed in the form of mean ± standard deviation (x ± s). One-Sample Kolmogorov–Smirnov test was used to determine whether the data are normally distributed or not. If the data were normal distributed, comparisons between the two groups were tested by paired or unpaired *t*-test. The comparisons among three or more groups were performed with one-way ANOVA and Tukey’s post hoc test. For data that were skewed distributed, paired sample Wilcoxon signed-rank test was used to make the comparison. Survival analysis was determined by Kaplan–Meier method and log-rank test. The association between circ_0000020 expression and pathological characteristics was assessed by Chi square test. The correlation between genes were analyzed by Pearson’s correlation coefficient. *P* < 0.05 was statistically meaningful.

## Results

### Circ_0000020 expressions in glioma and its correlation with clinicopathological characteristics

**Fig. 1:**
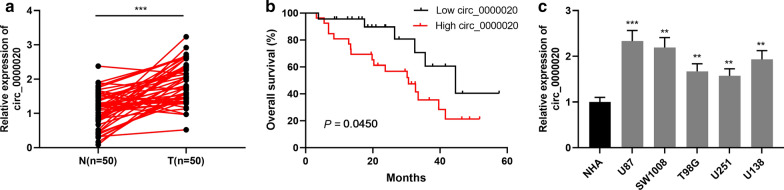
Circ_0000020 expression was upregulated in glioma tissues and cells. **a** qRT-PCR was used to detect the expression of circ_0000020 in 50 cases of gliomas and adjacent brain tissues. **b** Kaplan–Meier survival analysis was used to analyze the prognosis of the 50 cases of glioma patients according to circ_0000020 expression level. **c** qRT-PCR was used to detect the expression of circ_0000020 in NHA and 5 kinds of glioma cells (U87, SW1008, T98G, U251 and U138). (n = 3). N: normal tissue; T: tumor tissue; Error bars represented the mean ± SD of at least three independent experiments; **P < 0.01 and ***P < 0.001

To investigate the expression of circ_0000020 in gliomas, qRT-PCR was employed to measure circ_0000020 expressions in cancer tissues and adjacent normal tissues from 50 glioma patients, the results of which manifested that, in most cases, circ_0000020 expression in glioma tissues was dramatically elevated as against in normal brain tissues, independent of histologic glioma subtype (Fig. 1a). Kaplan–Meier analysis and log-rank test showed that patients with higher circ_0000020 (n = 27) expression had a significantly poorer overall survival rate compared to patients with lower circ_0000020 expression (n = 23) (*P *= 0.0450) (Fig. 1b). Additionally, Chi square test was adopted to analyze the relationship between the expression of circ_0000020 and clinical pathological indicators, and the results implied that circ_0000020 high expression was remarkably correlated with advanced WHO grade (III–IV) and larger tumor size (Table 2, *P* < 0.05). Besides, qRT-PCR was adopted to detect circ_0000020 expressions in NHA and 5 glioma cell lines. As shown, compared with in NHA cells, circ_0000020 expression in five glioma cells was observably up-regulated (Fig. 1C, Additional file 1: Figure S1). These data hinted that circ_0000020 exerted an oncogenic role in gliomas.

**Table 2:** Correlation between clinicopathological characteristics and hsa_circ_0000020 expression levels in glioma patients

Characteristics	Number of patients	hsa_circ_0000020 expression		*χ*^*2*^	*P* value
		Low (n)	High (n)		
Gender					
Male	29	11	18	1.8098	0.1785
Female	21	12	9		
Age (years)					
< 45	22	12	10	1.1549	0.2825
≥ 45	28	11	17		
Family history of cancer					
Yes	18	10	8	1.0338	0.3093
No	32	13	19		
Tumor size (cm)					
< 5	19	13	6	6.2019	*0.0128*
≥ 5	31	10	21		
WHO grade					
I–II	17	13	4	9.6275	*0.0019*
III–IV	33	10	23		
Tumor location					
Supratentorial	27	10	17	1.8983	0.1683
Infratentorial	23	13	10		
Peritumoral Brain Edema (cm)					
≥ 1	24	14	10	2.8263	0.0927
< 1	26	9	17		
Histological type					
OT	8	5	3	1.7340	0.4202
AT	35	16	19		
Other	7	2	5		

OT: Oligoastrocytic type; AT: Astrocytic type; Data in italics indicates statistical significance at *P *< 0.05

### Effects of circ_0000020 on cell proliferation, migration and invasion of glioma cells

**Fig. 2:**
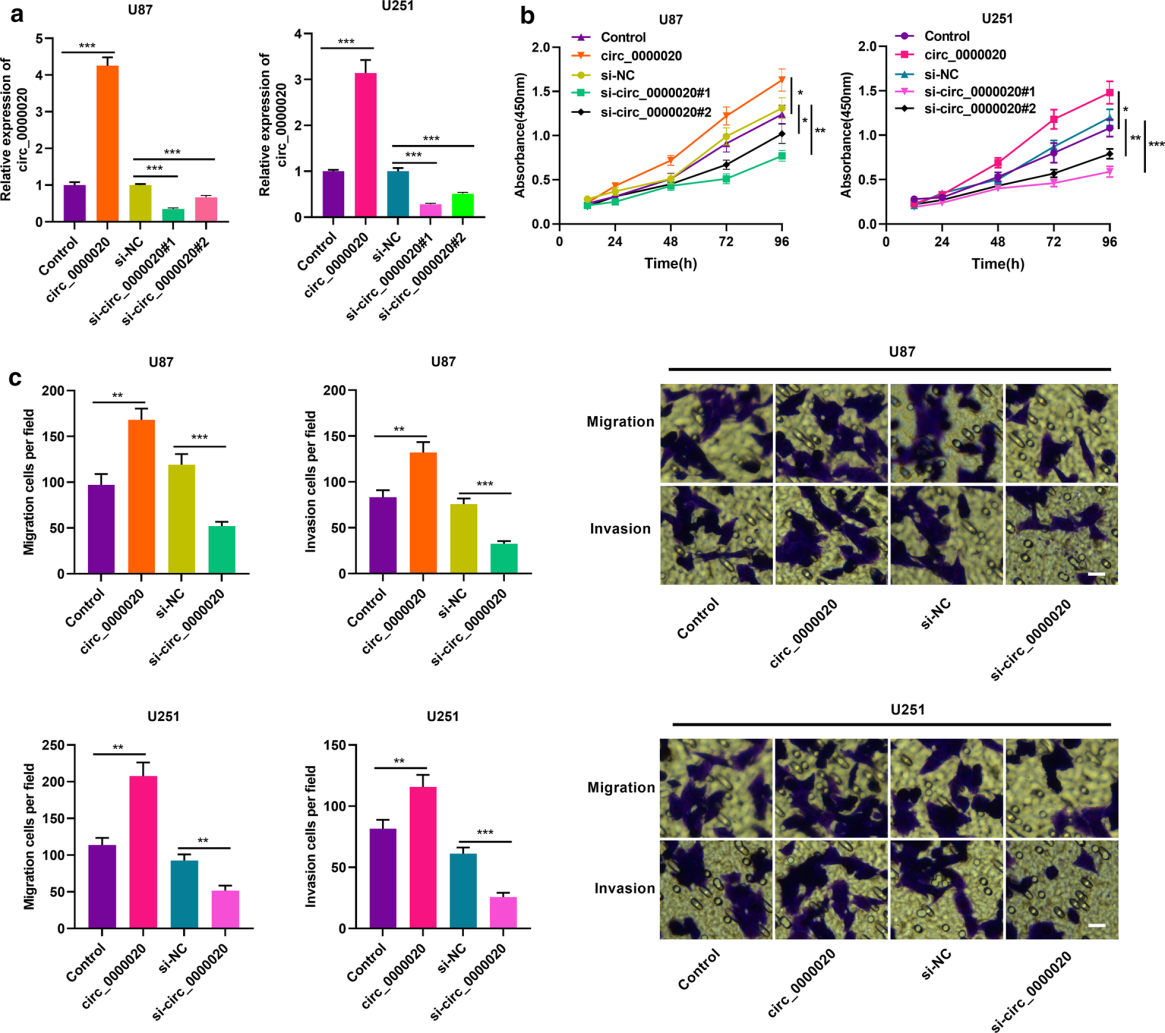
Circ_0000020 was involved in promoting the proliferation, migration and invasion of glioma cells. **a** Empty vector, circ_0000020 overexpression plasmid, si-NC and si-circ_0000020 were transfected into U87 and U251 cell line. The expression of circ_0000020 was detected by qRT-PCR 48 h after the transfection. (n = 3). **b** 48 h after the transfection, CCK-8 assay was used to detect cell proliferation after knockdown or overexpression of circ_0000020. (n = 3). **c** Transwell assay was used to detect cell migration and invasion after the knockdown or overexpression of circ_0000020 (magnification: ×400). (n = 3). si-NC: siRNA normal control, si-circ_0000020: siRNA against circ_0000020, Vector: pcDNA empty vector, circ_0000020: pcDNA-circ_0000020, Error bars represented the mean ± SD of at least three independent experiments; *P < 0.05, **P < 0.01 and ***P < 0.001

Next, we investigated the role of circ_0000020 in modulating the malignant phenotypes of glioma cells. We selected U87 and U251 cell lines to construct the circ_0000020 knockdown models and overexpression models (Fig. 2a). Notably, the siRNA targeting circ_0000020 effectively down-regulated the expression of circ_0000020 in U87 and U251 cells, and didn’t repress DDI2 (whose pre-mRNA probably gives rise to circ_0000020) expression (Additional file 1: Figure S2). CCK-8 assay was performed to detect glioma cell proliferation. As shown, compared with the control group, knocking down circ_0000020 dramatically inhibited glioma cell proliferation, while overexpression of circ_0000020 remarkably promoted glioma cell proliferation (Fig. 2b). In addition, compared with si-circ_0000020#2, si-circ_0000020#1 not only had a higher knockdown efficiency, but also more significantly inhibited the viability of glioma cells. Therefore, si-circ_0000020#1 has been used in subsequent experiments. Subsequently, through Transwell experiments, it was revealed that knockdown of circ_0000020 remarkably blocked cell migration and invasion, and overexpression of circ_0000020 exerted the opposite effects (Fig. 2c). The above data indicated that circ_0000020 participated in promoting the proliferation, migration and invasion of glioma cells.

### Circ_0000020 could target miR-142-5p

**Fig. 3:**
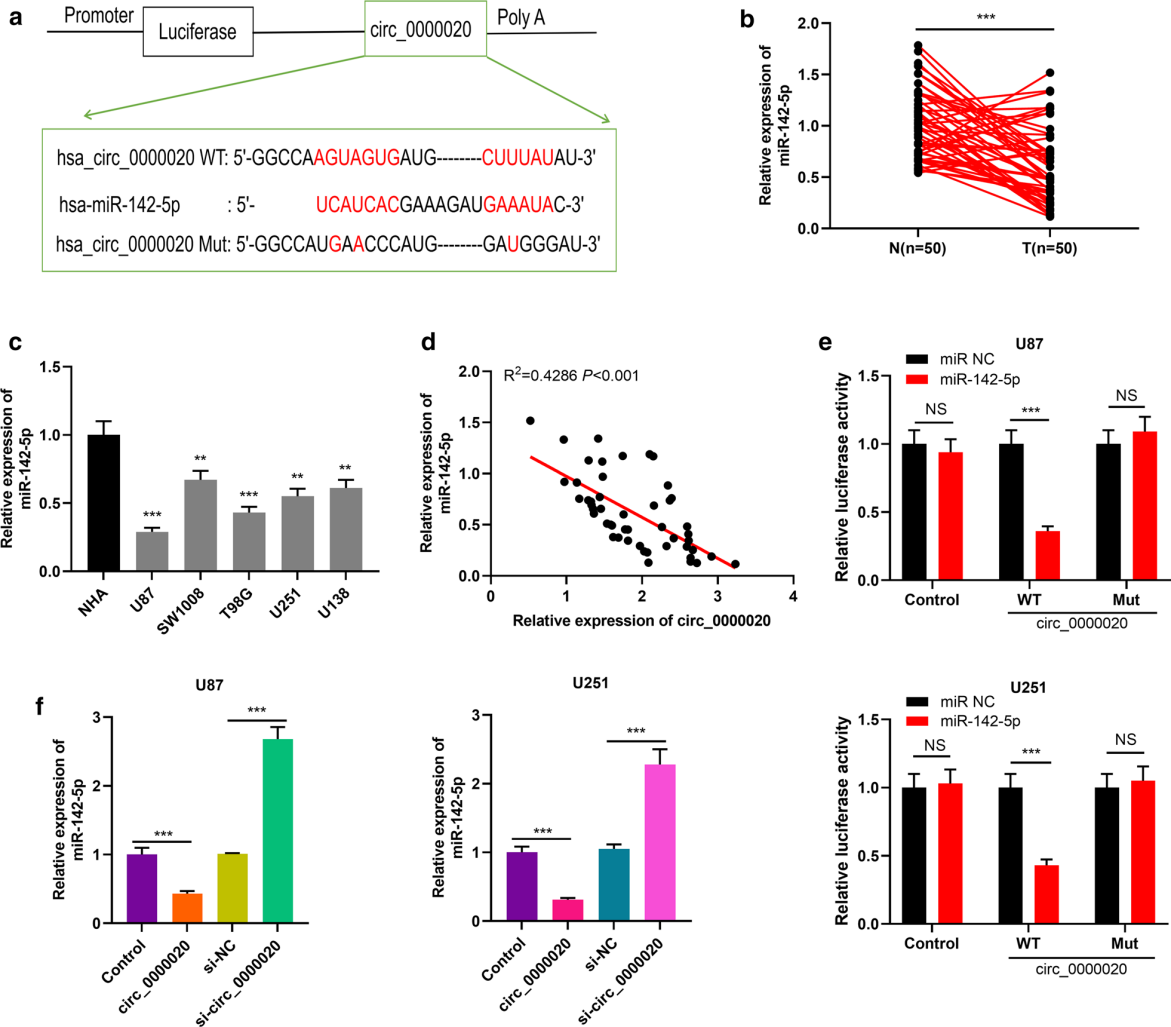
Circ_0000020 targeted miR-142-5p in glioma. **a** The potential targets of circ_0000020 were predicted by bioinformatics analysis, and circ_0000020 contained a potential binding site of miR-142-5p. **b** The expression of miR-142-5p in glioma tissues and normal tissue adjacent to tumor was detected by qRT-PCR. **c** qRT-PCR was used to detect the expression of miR-142-5p in NHA and 5 kinds of glioma cells (U87, SW1008, T98G, U251 and U138). (n = 3). **d** Person’s correlation coefficient was used to analyze the correlation between circ_0000020 and miR-142-5p expressions in glioma tissues. **e** WT-circ_0000020 reporter, Mut-circ_0000020 reporter, together with miR-142-5p mimics or miR NC, were co-transfected into U87 and U251 cells, respectively, and dual-luciferase gene reporter assay was used to validate the predicted binding site between circ_0000020 and miR-142-5p. (n = 3). **f** qRT-PCR was used to detect miR-142-5p expression in glioma cells after knockdown or overexpression of circ_0000020. (n = 3). N: normal tissue; T: tumor tissue; Control: pmirGLO-control, WT-circ_0000020: WT-pmirGLO-circ_0000020, Mut-circ_0000020: Mut-pmirGLO-circ_0000020, miR-142-5p: miR-142-5p mimics, miR NC: miR negative control, Error bars represented the mean ± SD of at least three independent experiments; **P < 0.01, ***P < 0.001, and NS: *P *> 0.05

To elucidate the downstream mechanism by which circ_0000020 regulates the phenotypes of glioma cells, bioinformatics analysis was performed using the CircInteractome database to predict the miRNA that could pair with circ_0000020 (Additional file 1: Figure S3). As shown, the potential binding site between circ_0000020 and miR-142-5p was predicted (Fig. 3a). Next, we detected miR-142-5p expression in glioma tissues and cell lines by qRT-PCR. As shown, miR-142-5p expression in glioma tissues was markedly lowered (Fig. 3b). Consistently, miR-142-5p expression in five glioma cell lines was also dramatically reduced compared with that in NHA cells (Fig. 3c). Then we analyzed the correlation between circ_0000020 and miR-142-5p expressions in 50 glioma tissues and Person’s correlation coefficient indicated that there was a negative correlation between circ_0000020 and miR-142-5p in glioma tissues (Fig. 3d). Then dual-luciferase reporter assay was utilized to verify the predicted binding site. The results demonstrated that miR-142-4p mimics could repress the luciferase activity of circ_0000020-WT reporter, but had no obvious effect on circ_0000020-MUT reporter (Fig. 3e). Moreover, qRT-PCR was utilized to detect the expression level of miR-142-5p after knockdown or overexpression of circ_0000020 in glioma cell lines. It was observed that miR-142-5p expression was elevated in U87 and U251 cells with circ_0000020 knockdown, but restrained in U87 and U251 cells with circ_0000020 overexpression (Fig. 3f). These data indicated that circ_0000020 targeted miR-142-5p and negatively modulated its expressions in glioma cells.

### MiR-142-5p could participate in inhibiting the proliferation and metastasis of glioma cells

**Fig. 4:**
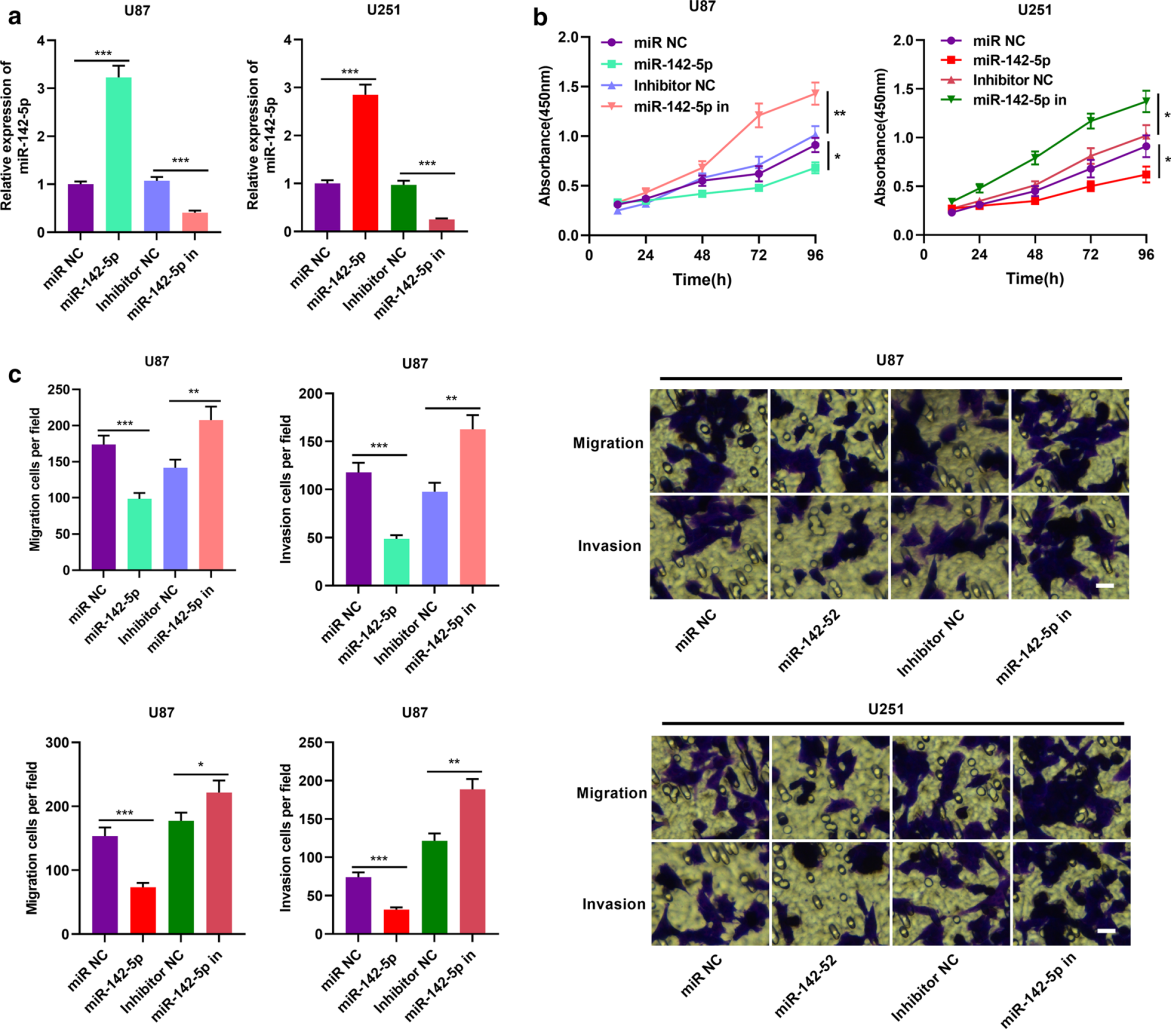
MiR-142-5p exerted tumor-suppressive effect in gliomas. **a** miR NC, miR-142-5p mimics, inhibitor NC and miR-142-5p inhibitors were transfected into U87 and U251 cells. The expression of miR-142-5p was detected by qRT-PCR 48 h after transfection. (n = 3). **b** After 48 h of transfection, CCK-8 assay was used to detect cell proliferation after the inhibition or overexpression of miR-142-5p. (n = 3). **c** Transwell assay was used to detect cell migration and invasion after inhibition or overexpression of miR-142-5p (magnification: ×400). (n = 3). Inhibitor NC: Inhibitor negative control, miR-142-5p in: miR-142-5p inhibitor, miR NC: miR negative control, miR-142-5p: miR-142-5p mimics, **P < 0.01 and ***P < 0.001

Then we transfected miR-142-5p inhibitors, mimics and their negative controls into U87 and U251 cells, and the successful transfection was ascertained by qRT-PCR, and the results showed that miR-142-5p expression was inhibited by the transfection of miR-142-5p inhibitors in U87 and U251 cells, but it was increase after the transfection of miR-142-5p mimics in U87 and U251 cells (Fig. 4a). Afterwards, we used CCK-8 and Transwell assays to detect cell proliferation, migration, and invasion. The results indicated that these malignant biological behaviors of glioma cells were dramatically facilitated after the transfection of miR-142-5p inhibitors, while the transfection of miR-142-5p mimics worked oppositely (Fig. 4b, c). These results implied that miR-142-5p inhibited the proliferation, migration and invasion of glioma cells.

### Circ_0000020 participated in regulating proliferation, migration, and invasion of glioma cells by targeting miR-142-5p

**Fig. 5:**
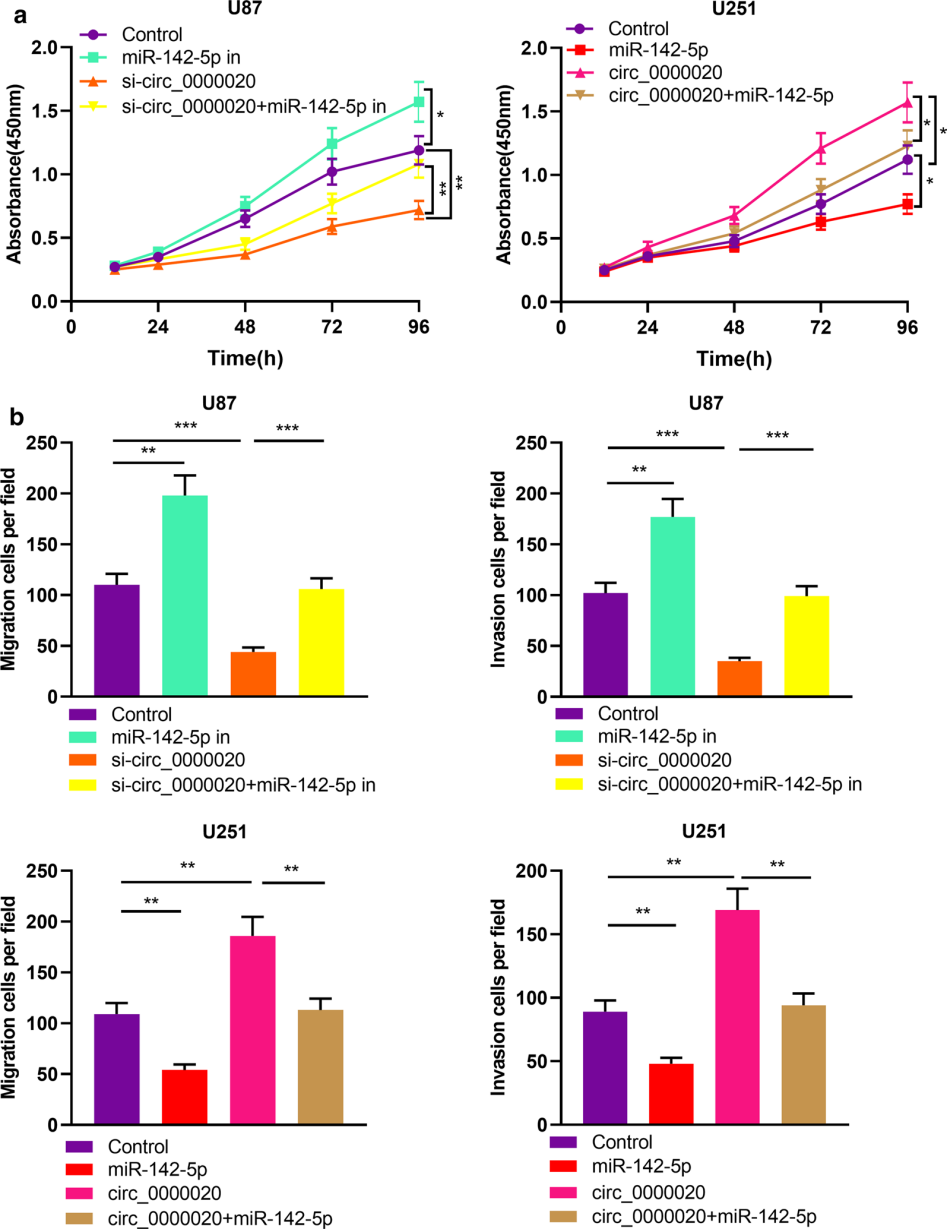
The effects of circ_0000020 on glioma cells was partly dependent on miR-142-5p. U87 cells were transfected with si-circ_0000020, miR-142-5p inhibitors, or si-circ_0000020 + miR-142-5p inhibitors; U251 cells were transfected with circ_0000020 overexpression plasmid, miR-142-5p mimics, or circ_0000020 overexpression plasmid + miR-142-5p mimics. **a**, **b** The proliferation, migration and invasion of were detected by CCK-8 assay (**a**) and Transwell assay (**b**), respectively. (n = 3). Control: negative control, miR-142-5p in: miR-142-5p inhibitor, si-circ_0000020: siRNA against circ_0000020, miR-142-5p: miR-142-5p mimics, circ_0000020: pcDNA-circ_0000020, Error bars represented the mean ± SD of at least three independent experiments; *P < 0.05, **P < 0.01 and ***P < 0.001

To further clarify the function of the circ_0000020/miR-142-5p axis in glioma progression, we then carried out “rescue experiments”. We found that inhibiting miR-142-5p partially reversed the inhibitory effect of knocking down circ_0000020 on the proliferation, migration, and invasion of U87 cells. The effect of overexpression of circ_0000020 on the proliferation, migration, and invasion of U251 cells was partly weakened by miR-142-5p mimics (Fig. 5a, b). These data indicated that circ_0000020 was involved in regulating the proliferation, migration, and invasion of glioma cells via targeting miR-142-5p.

### Circ_0000020/miR-142-5p axis regulated PIK3CA expression

**Fig. 6:**
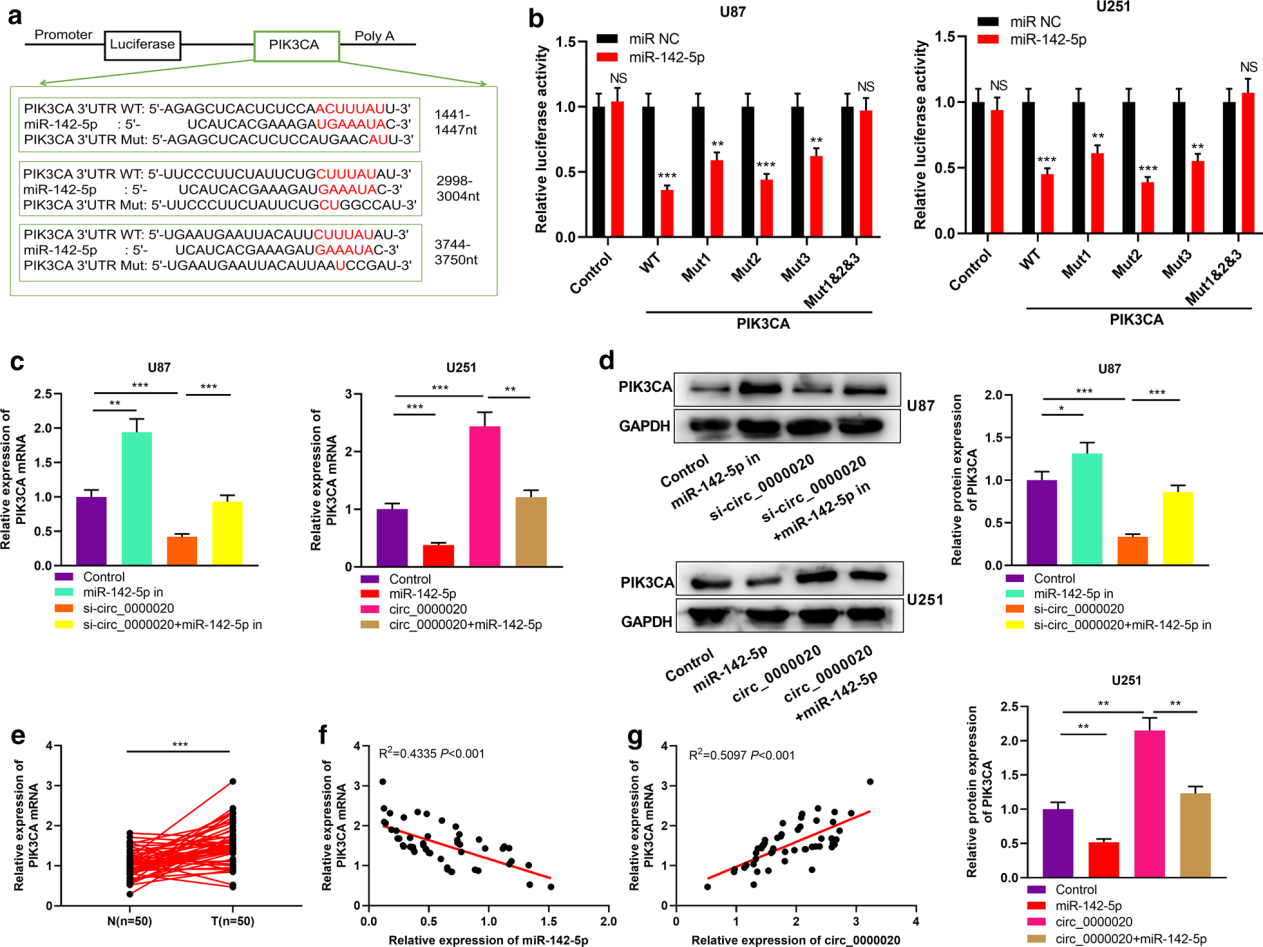
PIK3CA was regulated by circ_0000020/miR-142-5p axis in glioma. **a** The 3′ UTR of PIK3CA contained 3 putative binding sites for miR-142-5p. **b** WT-PIK3CA or Mut-PIK3CA reporters (Mut1, Mut2, Mut3 and Mut1&2&3), together with miR-142-5p or miR NC, were co-transfected into U87 and U251 cells, respectively, and dual-luciferase assay was used to verify the targeting relationship between PIK3CA and miR-142-5p in gliomas. (n = 3). **c**, **d** U87 cells were transfected with miR-142-5p inhibitors, si-circ_0000020, si-circ_0000020 + miR-142-5p inhibitors; U251 cells were transfected with miR-142-5p mimics, circ_0000020 overexpression plasmid, circ_0000020 overexpression plasmid + miR-142-5p inhibitors. Then the expression of PIK3CA was detected by qRT-PCR (**c**) and Western blot (**d**), respectively. (n = 3). **e** qRT-PCR was used to detect the expression of PIK3CA in 50 cases of gliomas and adjacent brain tissues. **f**, **g** Person’s correlation coefficient was used to analyze the correlations between PIK3CA expression and circ_0000020 or miR-142-5p expression in glioma tissues, respectively. WT-PIK3CA: WT-pmirGLO-PIK3CA 3′ UTR, Mut1-PIK3CA: Mut1-pmirGLO-PIK3CA 3′ UTR, Mut2-PIK3CA: Mut2-pmirGLO-PIK3CA 3′ UTR, Mut3-PIK3CA: Mut3-pmirGLO-PIK3CA 3′ UTR, Mut1 & 2 & 3-PIK3CA 3′UTR: Mut1 & 2 & 3-pmirGLO-PIK3CA, N: normal tissue; T: tumor tissue; GAPDH: glyceraldehyde-3-phosphate dehydrogenase, Error bars represented the mean ± SD of at least three independent experiments; *P < 0.05, **P < 0.01, ***P < 0.001 and NS: *P *> 0.05

After confirming that circ_0000020 could regulate miR-142-5p expression, we then explored the downstream targets of miR-142-5p. We searched for the candidate targets of miR-142-5p with StarBase, miRDB and TargetScan database, the findings of which depicted that miR-142-5p could probably target the PIK3CA (Fig. 6a, Additional file 2). Then we verified the targeting relationship between miR-142-5p and PIK3CA through dual-luciferase assay (Fig. 6b). Additionally, after the transfection of miR-142-5p inhibitors and mimics, the expression levels of PIK3CA mRNA and protein in glioma cells were up-regulated and down-regulated, respectively, and the opposite effects could be observed after circ_0000020 knockdown and overexpression; moreover, we also found that miR-142-5p inhibition partially reversed the down-regulation of PIK3CA mRNA and protein expressions induced by knocking down circ_0000020; miR-142-5p overexpression partly attenuated the up-regulation of PIK3CA mRNA and protein expressions due to overexpression of circ_0000020 (Fig. 6c, d). Besides, we also verified with qRT-PCR that PIK3CA mRNA expression was markedly elevated and positively correlated with circ_0000020 expression in glioma tissues and negatively correlated with miR-142-5p expression (Fig. 6e–g). The above results suggested that PIK3CA was a target of miR-142-5p in glioma and was positively regulated by circ_0000020.

### Overexpression of PIK3CA reversed the effect of circ_0000020 knockdown on glioma cells

In order to further verify the role of PIK3CA in the process of circ_0000020 promoting the development of glioma, siRNA against PIK3CA was transfected into U251 cell line with circ_0000020 overexpression. As shown, the promoting effects of circ_0000020 overexpression on PIK3CA expression in U251 cells were blocked by the co-transfection with si-PIK3CA (Additional file 1: Figure S4A, B). Consistently, the enhanced proliferation, migration and invasion of U251 cells due to circ_0000020 overexpression were also reversed by PIK3CA knockdown (Additional file 1: Figure S4C, D). These results suggested that the oncogenic properties of circ_0000020 were dependent on PIK3CA.

### Knockdown of circ_0000020 inhibited glioma growth and metastasis in vivo

**Fig. 7:**
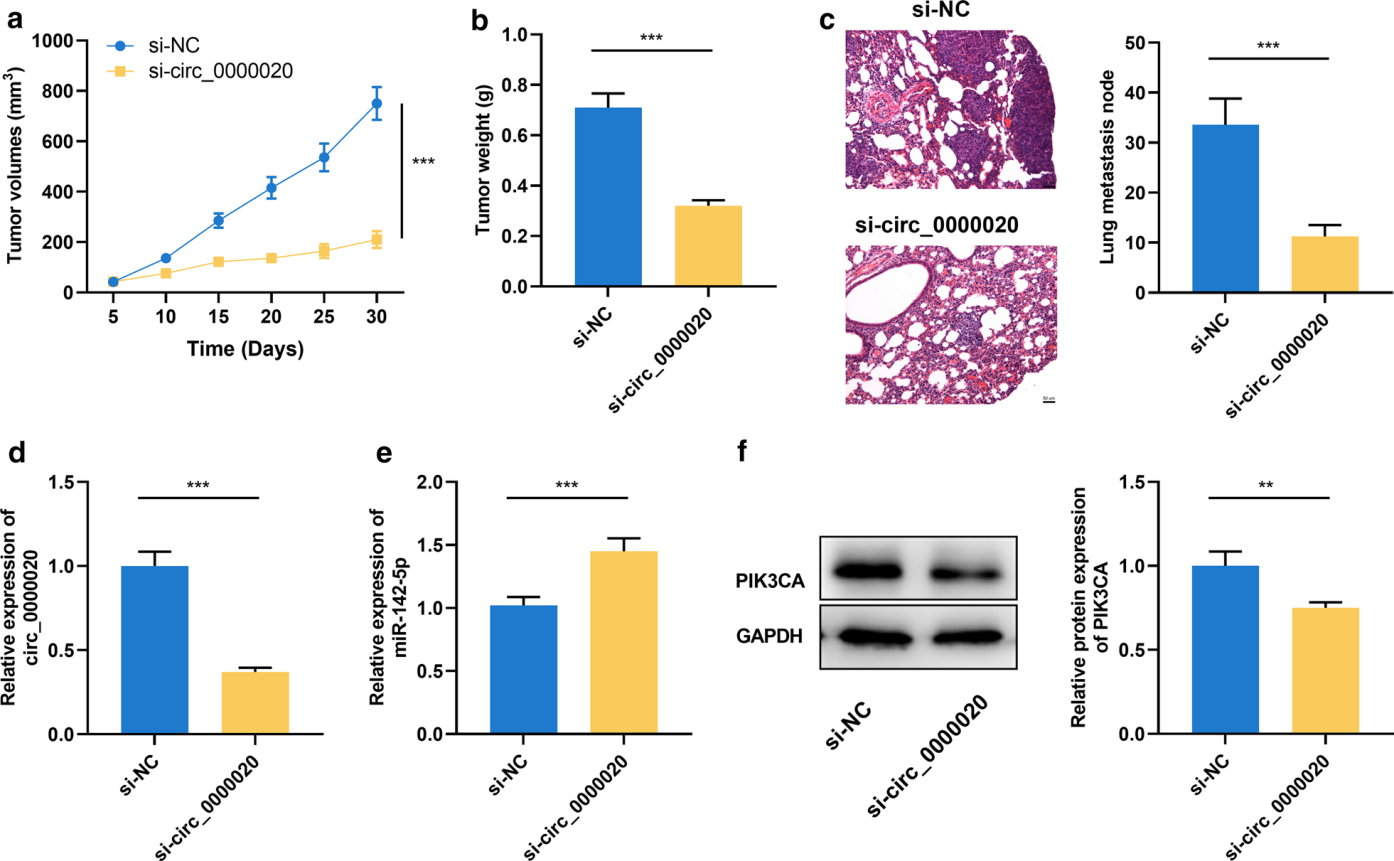
Knockdown of circ_0000020 inhibited glioma growth in vivo. **a**, **b** U87 cells transfected with si-circ_0000020 or si-NC were used for tumor xenograft assay. The tumor volume and weight in vivo was measured. **c** HE staining was used for evaluating lung metastasis of the mice. **d**, **e** The expressions of circ_0000020 and miR-142-5p in tumor tissues of the mice were examined by qRT-PCR. **f** The expression of PIK3CA in tumor tissues of the mice was examined by Western blot. si-NC: siRNA normal control, si-circ_0000020: siRNA against circ_0000020, GAPDH: glyceraldehyde-3-phosphate dehydrogenase, Error bars represented the mean ± SD of at least three independent experiments; ***P < 0.001

In addition to in vitro assays, xenograft tumor models were established in vivo in order to confirm the role of circ_0000020 in glioma growth and metastasis. U87 cells with circ_0000020 knockdown was subcutaneously injected into the nude mice. The result showed that compared with in the si-NC group, the size of tumors in mice in si-circ_0000020 group was significantly smaller (Fig. 7a). Moreover, the tumor weight in the si-circ_0000020 group were also markedly reduced compared with the si-NC groups (Fig. 7a, b). Additionally, after the U87 cells were injected into the mice via caudal vein, 2 weeks later, HE staining of lung sections revealed that knockdown of circ_0000020 in U87 cells markedly inhibited lung metastasis in mice (Fig. 7c). Furthermore, qRT-PCR was used to examined the expression of circ_0000020 in excised tumor tissues, and the result showed that the expression level of circ_0000020 was remarkably decreased, and miR-142-5p expression was significantly increased in si-circ_0000020 group compared with the si-NC group (Fig. 7d, e). Western blot showed that the protein expression of PIK3CA was remarkably decreased in si-circ_0000020 group compared with the si-NC group (Fig. 7f). These data further validated that that circ_0000020 regulated glioma progression via regulating miR-142-5p and PIK3CA.

## Discussion

In recent years, a large amount of endogenous circRNA has been detected in a variety of cells, which becomes a research hotspot in the field of non-coding RNA after miRNA and lncRNA [21–23]. CircRNA is abnormally expressed in many tumors and is involved in tumorigenesis and cancer progression [24–26]. Accumulating studies reveal that circRNA is abnormally expressed in gliomas and regulates the biological behaviors of glioma cells. For instance, circ_0001649 expression is reduced in gliomas and it exerts tumor-suppressive effects [8]. CircNFIX expression is upregulated in gliomas and it promotes cancer progression [27]. Circ_000173 facilitates the proliferation and invasion of glioblastoma cells via the miR-326/Wnt7B axis [28]. In this work, we observed that circ_0000020 expression in glioma tissues and cell lines was significantly up-regulated. It is reported that DDI2 is an oncogene [29], and circRNA’s enrichment is related to the expression level of precursor mRNA [30]. Therefore, the increase of circ_0000020 expression in glioma tissues and cell lines may be related to the expression of oncogene DDI2, which remains to be validated in the future. Furthermore, we demonstrated that circ_0000020 high expression was interrelated with higher WHO grade and larger tumor size. These results imply that circ_0000020 is promising to be an indicator to evaluate the prognosis of glioma patients. In addition, our functional experiments affirmed that overexpression of circ_0000020 could markedly expedite the proliferation and metastasis of glioma cells, while its knockdown could inhibit the malignant phenotypes of glioma cells, suggesting it is a potential therapy target for gliomas.

MiRNA negatively modulates the expressions of target genes via targeting the 3′-UTR of mRNA, thereby regulating the functions of various cells [31, 32]. MiR-142-5p expression is elevated and exerts an oncogenic role in colorectal cancer and renal cell carcinoma [13, 33]. Besides, miR-142-5p is low-expressed in pancreatic cancer, gastric cancer, and hepatocellular carcinoma, and functions as a tumor-suppressor [14, 34–36]. Also, previous studies have reported that miR-142-3p can remarkably inhibit glioma cell migration and invasion [15]. In this work, we confirmed that miR-142-5p expression was also reduced in glioma tissues and cells, and overexpression of miR-142-5p remarkably impeded the proliferation, migration, and invasion of glioma cells. After miR-142-5p expression was inhibited, the malignancy of glioma cells was significantly enhanced. These data verified that miR-142-5p suppressed the progression of glioma.

CircRNAs can function as competitive endogenous RNA (ceRNA) or miRNA molecular sponge, and adsorb miRNA to exert biological functions. For instance, circ_0007534 regulates the proliferation and migration of glioma cells via adsorbing miR-761 [37]; circ_0014359 is highly expressed in glioma tissues and functions as a miR-153 sponge to facilitate cancer progression [38]. In CircInteractome database, we found that multiple miRNAs could probably be targeted by circ_0000020, among which many miRNAs have been reported to be involved in the development of glioma. This implies that circ_0000020 plays an important role in glioma progression. In this work, we identified a binding site between circ_0000020 and miR-142-5p. We also proved that miR-142-5p mimics partly abolished the promotion of glioma cell proliferation and metastasis due to overexpression of circ_0000020. The inhibitory effect of knocking down circ_0000020 on the proliferation and metastasis of glioma cells was partially alleviated by miR-142-5p inhibitors. These results indicated that circ_0000020 could participate in regulating the proliferation, migration and invasion of glioma cells by adsorbing miR-142-5p.

PIK3CA, the activator of PI3K/AKT signaling, is expressed in normal human brain, lung, mammary gland, gastrointestinal tract, cervix, ovary and other tissues. PIK3CA is an oncogene that regulates somatic cell proliferation, differentiation, survival and many other important physiological functions [18]. Previous studies indicate that PIK3CA is frequently mutated in human solid tumors like head and neck squamous cell carcinoma, esophageal cancer, squamous cell lung cancer and NSCLC, which suggests its importance in the tumorigenesis of cancers [39–42]. PI3K/AKT signaling promotes cell growth and survival, and studies demonstrate that the expression of PI3K/AKT is up-regulated in glioma, which is consistent with circ_0000020 expression. In addition, dysregulation of PIK3CA is also a usual event during cancer progression, and it is regulated by multiple miRNAs. For example, miR-375 inhibits the development of osteosarcoma via targeting PIK3CA [43]; In liver cancer, miR-124 impedes the proliferation of cancer cells via targeting PIK3CA [44]. It is reported that PIK3CA is a target of miR-142-5p in non-small cell lung cancer, gastric cancer, and pancreatic cancer [45–47]. In this work, it was demonstrated that miR-142-5p could negatively regulate PIK3CA mRNA and protein expressions in glioma cells. What’s more, circ_0000020 could positively regulate PIK3CA mRNA and protein expressions. Intriguingly, miR-142-5p overexpression could partially reverse the promoting effect of circ_0000020 on the expression of PIK3CA. Also, PIK3CA knockdown could partially reverse the promoting effect of circ_0000020 overexpression on proliferation, migration and invasion. It is worth noting that miR-142-5p can inhibit the development of glioma by regulating other genes. For example, miR-142-5p inhibits the proliferation and invasion of glioma cells by targeting Sema3C [48]. Furthermore, miR-142-5p inactivates Wnt/β-catenin signaling via targeting Wnt3a, thus regulating the stem cell-like traits of glioma cells [49]. These studies suggest that circ_0000020/miR-142-5p can probably regulate glioma progression via other downstream mechanisms, besides PIK3CA.

## Conclusion

This study confirms that circ_0000020 is significantly overexpressed in glioma tissues and cell lines. The high expression level of circ_0000020 is closely related to advanced WHO grade and larger tumor size. In addition, we find that circ_0000020 promotes the proliferation and metastasis of glioma cells by regulating miR-142-5p/PI3KCA axis. These findings reveal the function and underlying mechanism of circ_0000020 in gliomas, suggesting that circ_0000020 may be a diagnostic marker and therapeutic target for gliomas.

## Supplementary information

**Additional file 1:** Additional figures.

**Additional file 2. **The targets of miR-142-5p in StarBase, miRDB and TargetScan databases.

**Additional file 3. **The original data of qRT-PCR, CCK-8 and luciferase reporter assay.

## Data Availability

Data used to support the results of this study can be obtained from the corresponding authors as required (Additional file 3).

## Abbreviations

circRNAs: Circular RNAs
miRNAs: MicroRNAs
PIK3CA: Phosphatidyl inositol kinase 3 catalytic subunit alpha
PI3Ks: Phosphatidylino-sitol 3-kinases
FBS: Fetal bovine serum
qRT-PCR: Quantitative reverse transcriptase PCR
ceRNA: Competitive endogenous RNA
NSCLC: Non-small cell lung cancer
