# Supplementary material for: Circ_0000020 elevates the expression of PIK3CA and facilitates the malignant phenotypes of glioma cells via targeting miR-142-5p
Source: Cancer Cell Int. 2021 Jan 28;21:79. doi: 10.1186/s12935-021-01767-5 (PMC7841906; doi:10.1186/s12935-021-01767-5)
Supplement: Supplementary file 1 — Additional file 1: Additional figures. [file 12935_2021_1767_MOESM1_ESM.docx]

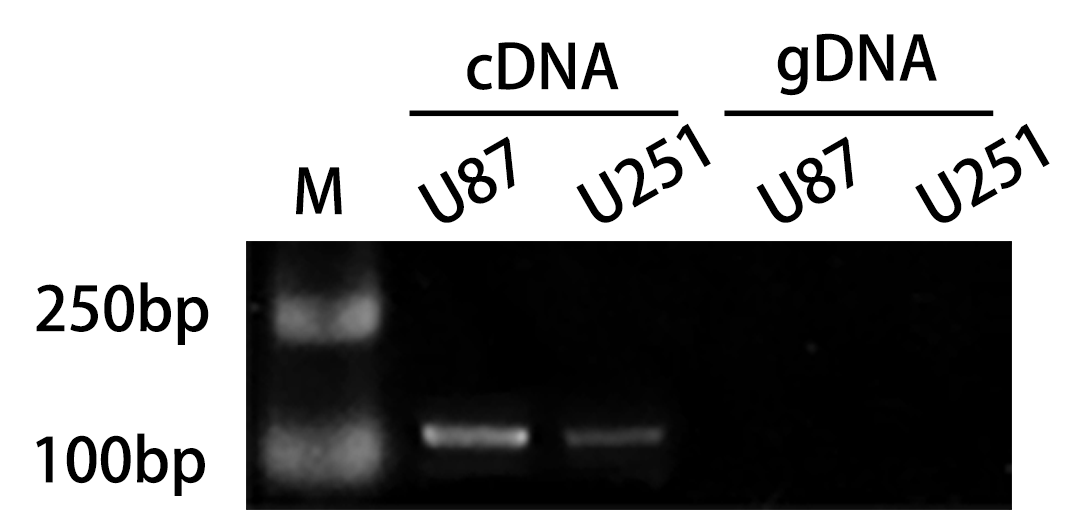


**Supplementary Figure 1 Agarose gel electrophoresis was performed to confirm the accuracy and specificity of primers for circ_0000020 using qRT-PCR products from U87 and U251 cells.**


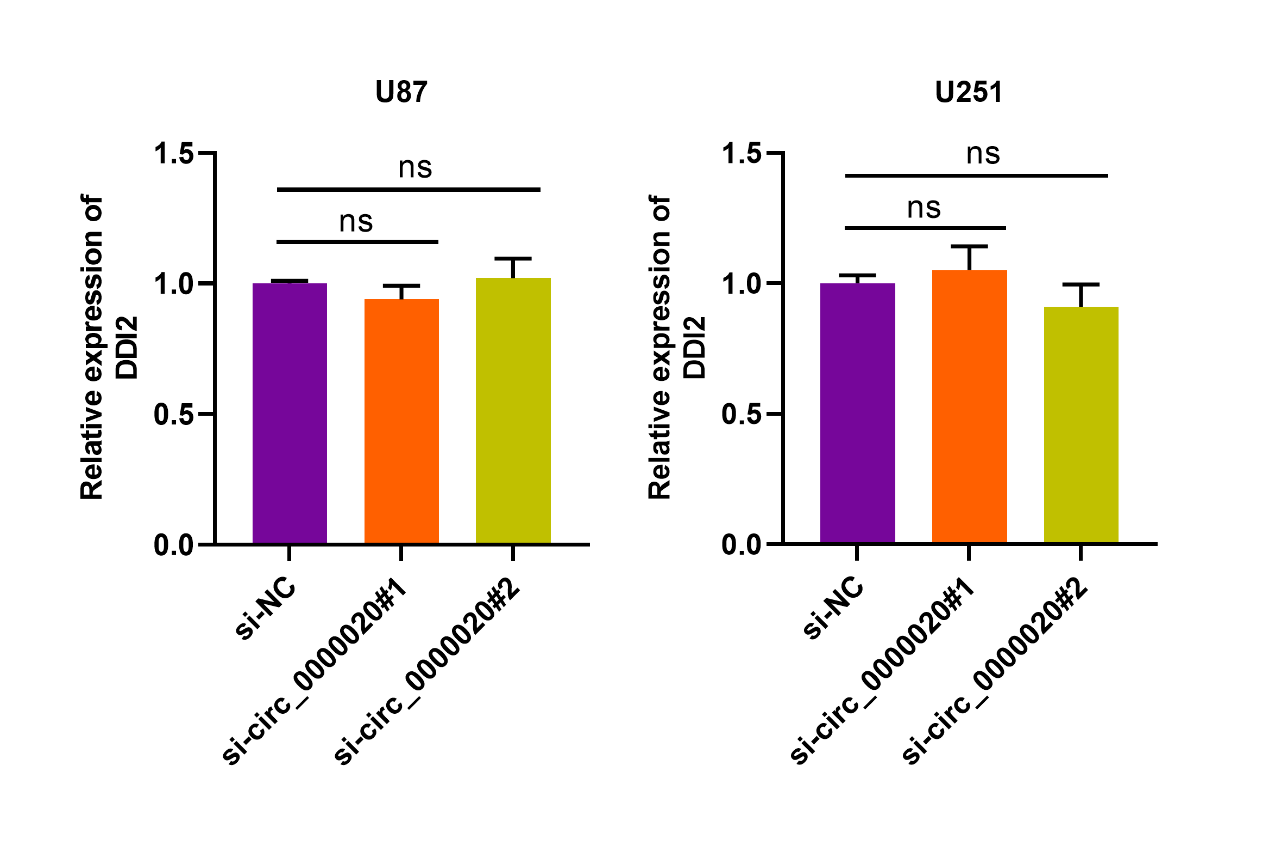


**Supplementary Figure 2 The expression level of DDI2 in U87 and U251 cell lines after the transfection with si-circ_0000020 or si-NC was examined by qRT-PCR.**


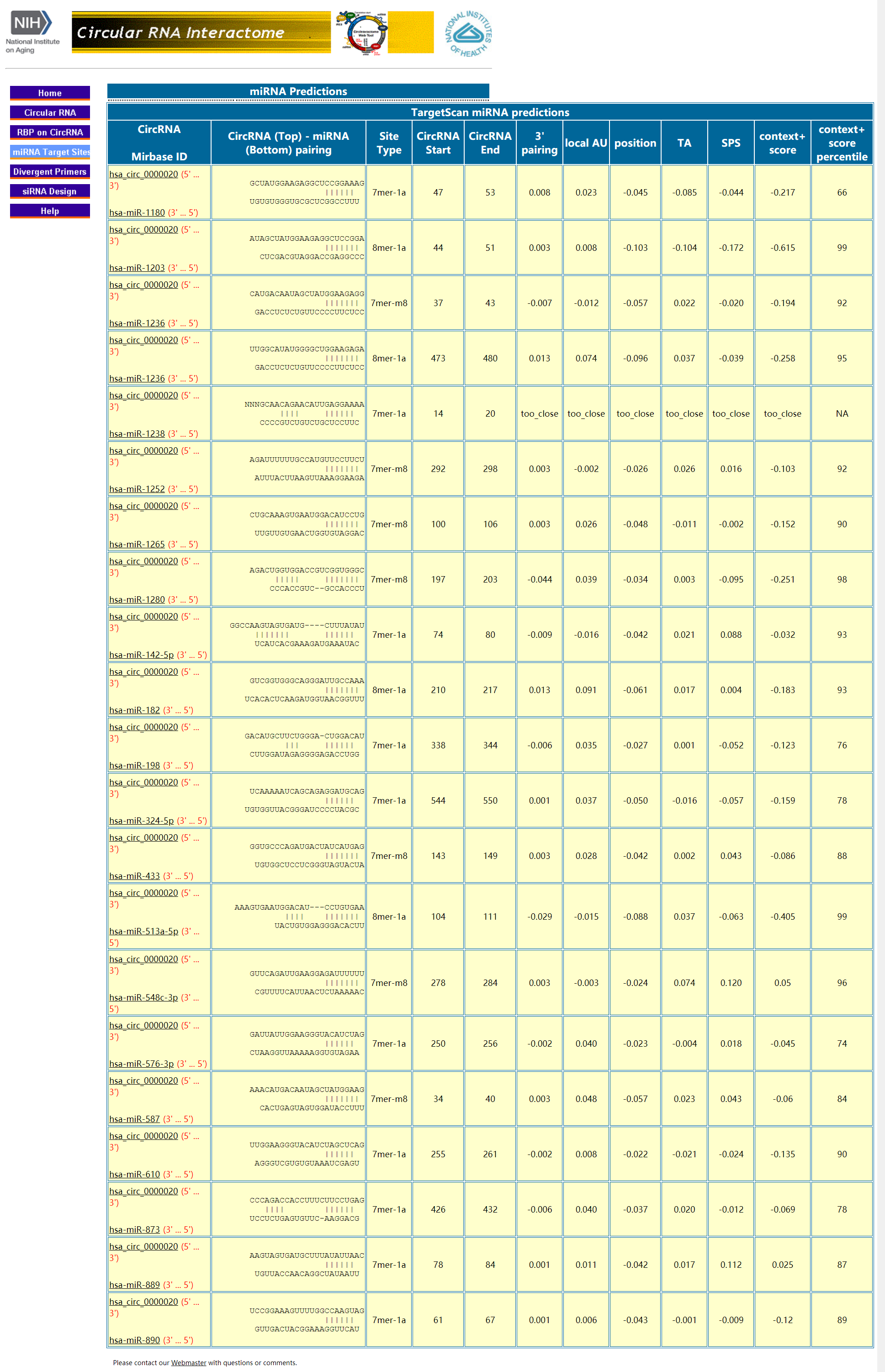


**Supplementary Figure 3 The potential miRNA targets of circ_0000020 predicted by CricInteractome database.**


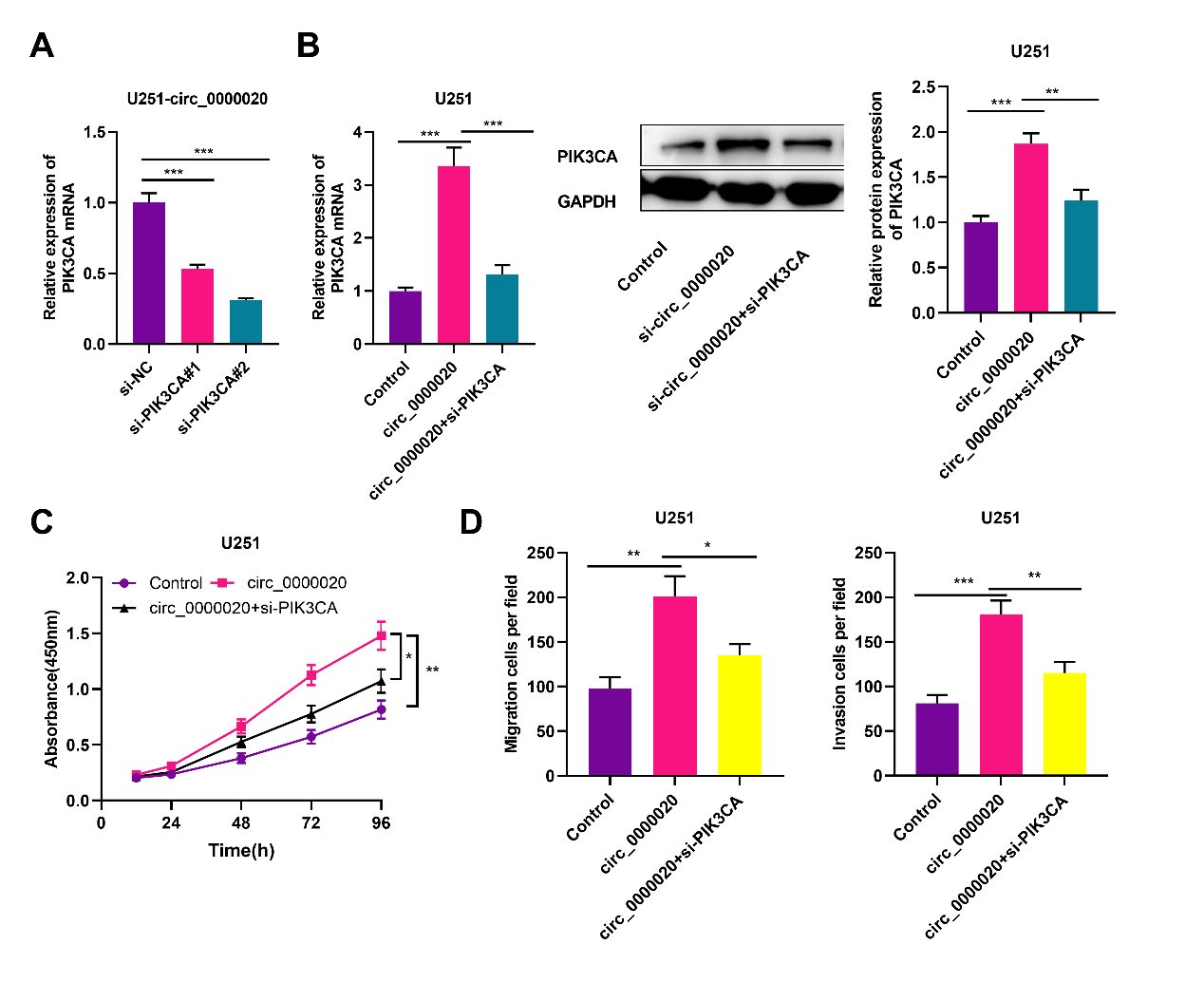


**Supplementary Figure 4. PIK3CA knockdown reversed the effect of circ_0000020 overexpression on glioma cells.**

A. siRNA against PIK3CA (si-PIK3CA#1 or si-PIK3CA#2) was transfected into U251 cell line with circ_0000020 overexpression, and the expressions of PIK3CA mRNA was detected by qRT-PCR.

B. The expressions of PIK3CA mRNA and protein were detected by qRT-PCR and Western blot assay, respectively.

C-D. The proliferation, migration and invasion of U251 cells con-transfected with si-circ_0000020 and si-PIK3CA were detected by CCK-8 assay and Transwell assay, respectively.

Control: negative control, circ_0000020: pcDNA-circ_0000020, si-PIK3CA: siRNA against PIK3CA, Error bars represented the mean ± SD of at least three independent experiments; * *P* < 0.05, ** *P* < 0.01 and *** *P* < 0.001
